# Genetic diversity and population structure of cowpea [*Vigna unguiculata* (L.) Walp.] accessions from Togo using SSR markers

**DOI:** 10.1371/journal.pone.0252362

**Published:** 2022-10-05

**Authors:** Yao Dodzi Dagnon, Koffi Kibalou Palanga, Damigou Bammite, Amy Bodian, Ghislain Comlan Akabassi, Daniel Foncéka, Koffi Tozo

**Affiliations:** 1 Laboratoire de Biotechnologies, Physiologie et Biologie Moléculaire Végétales, Faculté des Sciences, Université de Lomé, Lomé, Togo; 2 Institut Supérieur des Métiers de l’Agriculture, Université de Kara (ISMA-UK), Kara, Togo; 3 Centre d’étude régional pour l’amélioration de l’adaptation à la sécheresse (CERAAS), Thiès, Sénégal; 4 UFR Biosciences, African Center of Excellence for Climate Change, Biodiversity and Sustainable Agriculture, Cocody, Abidjan, Cote d’Ivoire; 5 Laboratory of Applied Ecology, University of Abomey Calavi, Abomey Calavi, Cotonou, Benin; North Dakota State University, UNITED STATES

## Abstract

Cowpea [*Vigna unguiculata* (L.) Walp.] is a crop with significant agronomic and nutritional value. In Togo, the crop is very appreciated by local people. It is the third food habit in Togo after maize and rice. However, several accessions of cowpea cultivated in Togo are now prone to extinction, creating a risk of genetic erosion. It is therefore urgent to assess the genetic diversity of accessions in order to set up a good conservation program. To achieve this, genetic diversity and phylogenetic relationships among 70 accessions of cowpea collected in the five (5) administrative regions of Togo were assessed using Simple Sequence Repeat (SSR) molecular markers. The twenty-eight SSR primers used in this study generated a total of 164 alleles with an average of 5.82 alleles per locus. Polymorphic Information Content (PIC) values ranged from 0.20 to 0.89 with an average value of 0.58. Population structure analysis using model-based revealed that the cowpea germplasm was grouped into two subpopulations. The analysis of molecular variance (AMOVA) revealed that 98% of genetic variation existed among accessions within regions. The fixation index (Fst) value, which was 0.069 was low, indicating relatively low population differentiation. The phylogenetic analysis grouped the 70 accessions into two main groups that can be further divided into four groups independent of their origins. This study provides a foundation for a Togolese cowpea germplasm conservation program and can serve for the selection of parental material for further studies aimed at the genetic improvement of local germplasm.

## Introduction

Cowpea [*Vigna unguiculata* (L.) Walp.] is an important food legume in developing countries of the tropics and subtropics, especially in Sub-Saharan Africa, Asia, and Central and South America [1, 2], and in some temperature area, including the Mediterranean region and the southern states of the USA [3, 4]. Its global annual production is 3.5 million metric tons, and Nigeria alone produced over 2.24 million metric tons on 2.52 million ha, followed by Niger, which produced 1.77 million metric tons on 5.57 million ha in 2017 [5]. Cowpea is commonly cultivated as a nutritious and highly palatable food source. The seed is reported to contain 24% crude protein, 53% carbohydrates, and 2% fat [6]. It is referred to as the ‘poor man’s meat’ because of its good protein quality and high nutritional value [7]. Besides, its hay is also useful in the feeding of animals during the dry season in many parts of West Africa [8, 9]. All parts of the cowpea are used for food. The leaves, green pods, green peas and dry grains are consumed as different dishes. Cowpea plays a very important subsistence role in the diets of many households in Africa [10]. It also has an economic value to the farming households since it is also a cash crop [11]. Besides, cowpea is a valuable component of farming systems in many areas because of its ability to restore soil fertility through nitrogen fixation for succeeding cereal crop grown in rotation with it [8, 12, 13].

Of late, the increase in world population and problems related to climatic variability has led to high demand of food. Unfortunately, in Togo for instance, many cowpea landraces are abandoned due to the farmer’s preference for variety presenting a relatively short development cycle, a higher productivity, a good market value and a good taste [14, 15]. The climatic variability makes farmers to select the landraces which have a short vegetative cycle [15]. All those facts lead to genetic erosion of the crop [2]. The main goal of cowpea breeding and genetic improvement programs around the world is to combine desirable agronomic traits such as time to maturity, photoperiod sensitivity, plant type and seed quality with resistance to the major biotic stresses threatening the crop production [9, 16].

Genetic diversity is the extent to which heritable material differs within a group of plants as a result of evolution, including domestication and plant breeding. Assessing the genetic diversity of cowpea germplasm is a prerequisite for effective breeding and germplasm conservation. Genetic studies of cowpea diversity have been carried out in several countries using DNA molecular markers such as random amplified polymorphic DNA (RAPD) [7, 17], amplified fragment length polymorphisms (AFLP) [18], restriction fragment length polymorphisms [19], inter-simple sequence repeat (ISSR) [4] and simple sequence repeat (SSR) [9, 20]. Of all these markers, SSR is the most widely used marker in genetic diversity analysis due to its multiallelic nature, high reproducibility, co-dominant inheritance, abundance and extensive genome coverage that has already been reported for crops like pigeon pea [21, 22] or rice [23–26]. The earliest use of SSR for assessing the genetic diversity of cowpea was conducted by Li et al. [20]. SSRs are also used to identify genotype, seed purity evaluation and variety protection, pedigree analysis and genetic mapping of simple and quantitative traits and marker-assisted selection breeding [9, 20, 27]. Prior to this study, there was no study conducted on genetic diversity of cowpea germplasm in Togo. The present study was, therefore, undertaken to provide a glance on the country cowpea germplasm genetic diversity based on SSR markers.

## Materials and methods

### Plant materials

Togo is a west-African country covering about 56,600 square kilometers and stretching from its 51 kilometers coastline in the Gulf of Guinea northward for about 515 kilometers between Ghana to the west and Benin to the east to its boundary with Burkina Faso in the north. The country is divided in five administrative regions namely Maritime, Plateaux, Centrale, Kara and Savanes and is under a general tropical climate with average temperatures ranging from 23°C on the coast to about 30°C in the northernmost regions. In the three northernmost regions, there is a single wet season occurring between May and November. In the south, there are two seasons of rain (the first between April and July and the second between September and November), even though the average rainfall is not very high [15].

As a crop cultivated across the whole country, the cowpea accessions were collected in the country’s five administrative region. The collection was done in collaboration with the Togolese Institute of Advices and Technical Supports (ICAT) technicians who are in charge of providing assistance and technical supports to farmers in order to help them maximize their crop production and by using the snowball method. Briefly, for the villages’ selection, firstly the villages producing cowpea and cowpea producer in each region were identified based on informations provided by ICAT technicians. Then in the first selected village of each region cowpea producers were asked to cite the villages known for cowpea production and those that have cultivars that were absent in their own village. The choice of the next village was done following the farmers recommendations after consulting the ICAT agents.

**Fig 1:**
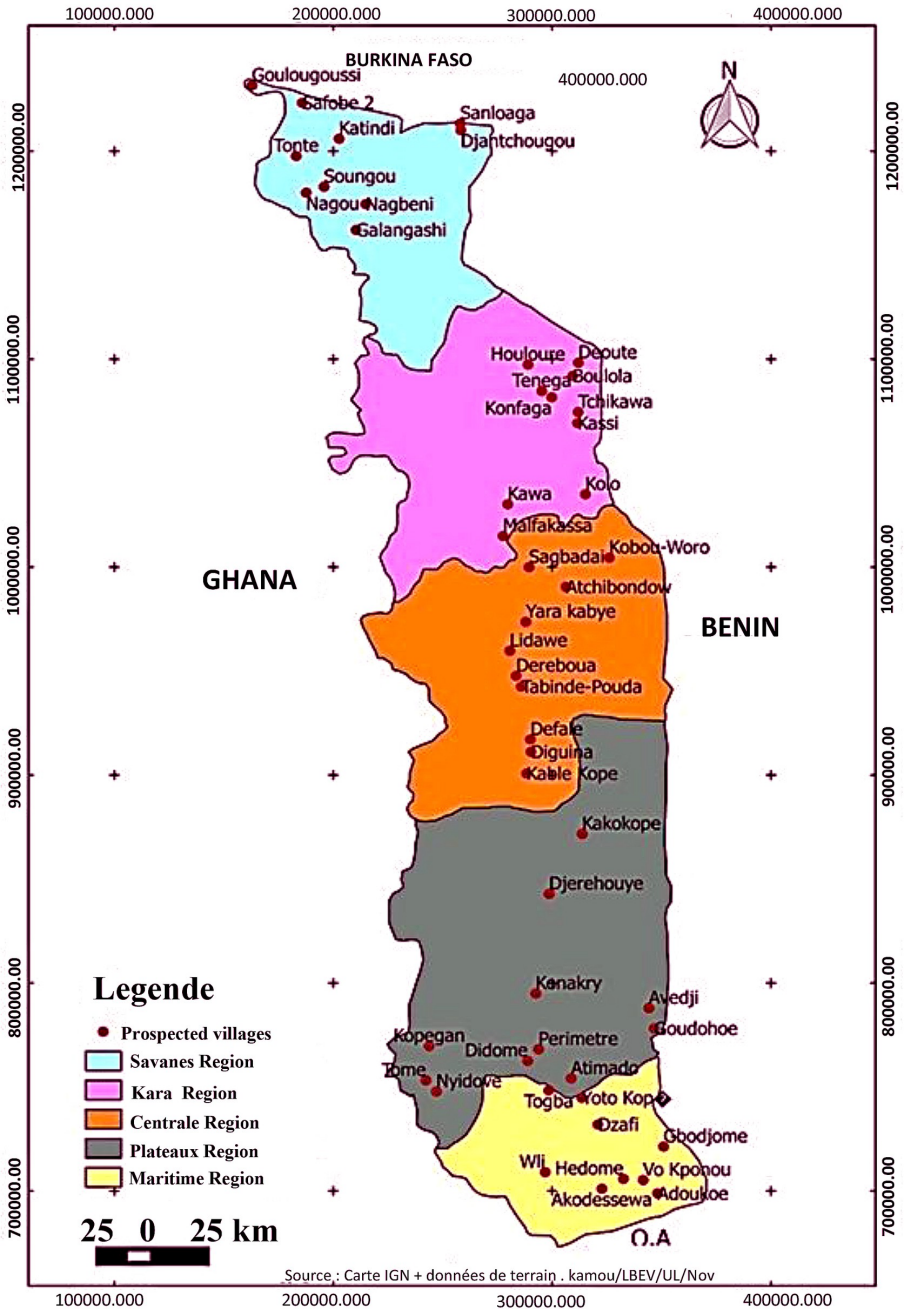
Map of geographic location of germplasm collection.

Using that method, a total of 70 cowpea accessions were collected from producers of 50 villages in the five regions of Togo between 2014 and 2016 (Fig 1, Table 1). Provenance, the local name of the seed and the villages’ coordinates were recorded. Among the 70 cowpea collected accessions, three are varieties listed in the national catalogue of species and varieties grown in Togo. These varieties are VITOCO and TVX bred by IITA-IBADAN, and VITA5 bred by the University of Ifê (Nigeria) and are widely cultivated in Togo. They were obtained from the Togolese Institute of Agriculture Research (ITRA). The 70 cowpea accessions represent the collection from all major growing areas of cowpea in Togo. Their local name and place of collection are provided in Table 1. In this study, all accessions from a region were considered as a population.

**Table 1:** List of the Cowpea accessions, characteristics and collection.

Accessions	Growth habit	Flower color	Seed size	Seed color	Status	Regions
Yélengo	Creeping	Purple	small	Beige red	Landrace	Centrale
Gbédéfouba	Creeping	Purple	small	Beige red	Landrace	Centrale
Guinsibibè	Creeping	White	big	white	Landrace	Centrale
Sotouboua	Creeping	White	big	white	Landrace	Centrale
Hèkou hèkou	Creeping	White	medium	white	Landrace	Centrale
Tchéwo	Creeping	White	small	white	Landrace	Centrale
Tchéwo koumoka	Creeping	White	small	white	Landrace	Centrale
Vitoco 2	Erected	Purple	small	white	Breeding Line	Centrale
Komi	Creeping	White	small	white	Landrace	Centrale
Vita 5	Creeping	White	small	white	Breeding Line	Centrale
Kétchéyi soukpèlo	Erected	Purple	small	purple red	Landrace	Kara
Kétchéyi Koussémo	Creeping	Purple	small	Red wine	Landrace	Kara
Kétchéyi	Erected	Purple	small	Burgundy purple	Landrace	Kara
Djodjowou	Creeping	White	big	white	Landrace	Kara
Koufaldo	Creeping	White	big	white	Landrace	Kara
Dapango kaga	Creeping	White	medium	white	Landrace	Kara
Dapango Koukpèto	Creeping	White	medium	white	Landrace	Kara
Kandjarga	Creeping	White	medium	Yellow sand	Landrace	Kara
Lamga	Creeping	White	small	white	Landrace	Kara
Simpayo	Creeping	White	small	white	Landrace	Kara
Tinkou	Creeping	White	small	white	Landrace	Kara
Sodjadéawoudadè	Semi erected	Purple	medium	Beige red	Landrace	Maritime
Togbéyi	Creeping	Purple	medium	Beige red	Landrace	Maritime
Amélassiwa	Semi erected	White	small	white	Landrace	Maritime
Dakarvi	Creeping	White	small	white	Landrace	Maritime
Kpédéviyi	Creeping	Purple	small	Beige red	Landrace	Maritime
Kpédévi	Creeping	Purple	small	Beige red	Landrace	Maritime
Téklikoé	Creeping	Purple	small	Rouge noir	Landrace	Maritime
Damadoami	Creeping	Purple	small	purple red	Landrace	Maritime
Itouloka	Creeping	Purple	small	Burgundy purple	Landrace	Maritime
Assiamaton	Semi erected	White	medium	white	Landrace	Maritime
Yéboua	Creeping	White	medium	white	Landrace	Maritime
Agnokoko	Creeping	White	small	white	Landrace	Maritime
Amélassiwa 2	Creeping	White	small	white	Landrace	Maritime
Gban molou	Creeping	White	small	white	Landrace	Maritime
Sakawouga	Creeping	Purple	medium	Reddish grey	Landrace	Plateaux
Ayi djin	Erected	Purple	medium	Beige red	Landrace	Plateaux
Tcharabaou djin	Creeping	Purple	medium	Red wine	Landrace	Plateaux
TVX	Erected	White	small	white	Breeding Line	Plateaux
Poli poli	Creeping	Purple	small	Beige red	Landrace	Plateaux
45 jours rouges	Erected	Purple	small	purple red	Landrace	Plateaux
Maca	Creeping	Purple	small	Red wine	Landrace	Plateaux
Kétchéyi 2	Semi erected	Purple	small	Burgundy purple	Landrace	Plateaux
Azangba	Erected	Purple	small	Burgundy purple	Landrace	Plateaux
Agamassikè	Creeping	White	medium	white	Landrace	Plateaux
Amélassiwa 3	Creeping	White	medium	white	Landrace	Plateaux
Sotoco	Creeping	White	medium	white	Landrace	Plateaux
Vitoco	Semi erected	White	medium	white	Breeding Line	Plateaux
Atakpamé	Creeping	White	medium	white	Landrace	Plateaux
Pamplovi	Creeping	White	small	white	Landrace	Plateaux
Siéloune	Semi erected	White	small	Yellow Gold	Landrace	Savannah
Malgbong bomoine	Semi erected	Purple	small	purple red	Landrace	Savannah
Esatoune	Creeping	Purple	small	Red wine	Landrace	Savannah
Simporé	Creeping	White	big	white	Landrace	Savannah
Atougbenda	Semi erected	White	medium	white	Landrace	Savannah
Bieng nomio	Creeping	White	medium	white	Landrace	Savannah
Golenga	Creeping	White	medium	white	Landrace	Savannah
Malgbong bopiel	Creeping	White	medium	white	Landrace	Savannah
Pélam	Creeping	White	medium	white	Landrace	Savannah
Alacante	Semi erected	White	medium	white	Landrace	Savannah
Toi	Semi erected	White	medium	white	Landrace	Savannah
Bieng oune	Creeping	White	small	white	Landrace	Savannah
Etougnognoli	Creeping	Purple	small	white	Landrace	Savannah
Etoukakali	Creeping	White	small	white	Landrace	Savannah
Gouarga	Creeping	White	small	white	Landrace	Savannah
Itouloka	Creeping	White	small	white	Landrace	Savannah
Kampirigbène	Semi erected	White	small	white	Landrace	Savannah
Natoguildjole	Creeping	White	small	white	Landrace	Savannah
Téléga	Semi erected	White	small	white	Landrace	Savannah
Toboni	Creeping	White	small	white	Landrace	Savannah

### DNA extraction

The DNA extraction and PCR amplification were done at the Centre d’Etude Régional pour l’Amélioration de l’Adaptation à la Sécheresse (CERAAS) in Senegal. The genomic DNA was extracted from a bulk of fresh leaf material of 21 day-old-plants of each of the 70 cowpea accessions following the mixed alkyl trimethyl ammonium bromide (MATAB) protocol described by Risterucci et al. [28]. The extracted DNA was quantified on 0.8% agarose gel in comparison to the bands of a Smart Ladder (MW-1700-10- Eurogentec) [29]. The working DNA concentration was then adjusted to 25 ng/μL.

### Polymerase chain reaction using SSR markers

A total of 28 polymorphic SSR markers were used to screen 70 cowpea DNA samples (Table 2). Those primers were selected after the screening of a set of three hundred of cowpea primers available at the CERAAS center. The forward and reverse primers for each of the 28 SSR markers (Table 2) were labeled at their 5’ end with fluorescent dyes to enable detection. The PCR reaction was conducted in a total volume of 10 μl, (5 μl of DNA and 5 μl of a PCR solution). The PCR solution was prepared with 55 μL of 10X buffer, 55 μL of dNTPs (200 μg), 22 μL of MgCl_2_ (0.5 mM), 9 μL of each primer (0.1 μM), 9 μL of IR dye (0.1 μM), 55 μL of Taq Polymerase 1U and 227 μL of ultrapure water. The PCR reaction was carried out in a 96-block thermal cycler (MWG AG biotech). The thermal cycling conditions were as follows: initial denaturation step at 94°C for 4 min followed by 26 cycles of denaturation (94°C) for 60 s, hybridization (50–55°C according to the primers) for 1 min, primer extension (72°C) for 1 min 15 seconds, followed by a final extension at 72°C for 7 min. After PCR, a 0.8% agarose gel was used to control the quality of the amplification products. The PCR plates were then covered with aluminum foil to prevent fluorochrome degradation and placed in a refrigerator for conservation purposes.

**Table 2:** Primer sequences of the 28 simple sequence repeat (SSR) markers used in this study [29].

N°	SSR name	Left sequence (5’ → 3’)	Right sequence (5’ → 3’)
1	MA 113	CACGACGTTGTAAAACGACTCGCACACAGATCCAACATT	CCTTATTTCTGGTGGGAGCA
2	MA 120	CACGACGTTGTAAAACGACCTTGGGGTGATGATGAAACC	AGGGGTGAAAAGTTGTCTTGC
3	SSR 6215	CACGACGTTGTAAAACGACGCTTCCCCGCTAGAATCTTT	GGTGCCAATGGATCAGGTAA
4	SSR 6217	CACGACGTTGTAAAACGACGGGAGTGCTCCGGAAAGT	TTCCCTATGAACTGGGAGATCTAT
5	SSR 6239	CACGACGTTGTAAAACGACCACTTTCTCCTAAGCACTTTTGC	AAGTGAAGCATCATGTTAGCC
6	SSR 6241	CACGACGTTGTAAAACGACCACTTTCTCCTAAGCACTTTTGC	TTGATGGAGTTCGCATCTTCT
7	SSR 6243	CACGACGTTGTAAAACGACGTAGGGAGTTGGCCACGATA	CAACCGATGTAAAAAGTGGACA
8	SSR 6245	CACGACGTTGTAAAACGACCGAACATGTTTTTGGTCACG	CTACAACCGCGTTAGCCTTC
9	SSR 6246	CACGACGTTGTAAAACGACTCTTGGGTCTCCAAAATCTGTAA	TTTCTATTGGGGTCCCCTTC
10	SSR 6288	CACGACGTTGTAAAACGACGATGTTGTAGCAGGCTAATTGGA	TGGCCAATTGTCCTAAGTTGA
11	SSR 6289	CACGACGTTGTAAAACGACCCCCCAAAGTTGATGAACAC	TTGATGGAGTTCGCATCTTCT
12	SSR 6304	CACGACGTTGTAAAACGACCTGGAACAAGTCGAGATGGAA	CCATCCCCCACCAAAAGT
13	SSR 6311	CACGACGTTGTAAAACGACATGCCATTGTTGAGTTGCTTT	AGGATGTTGTAGCAGGCTAATTG
14	SSR 6323	CACGACGTTGTAAAACGACCAAAGGGTCATCAGGATTGG	TTTAAGCAGCCAAGCAGTTGT
15	SSR 6421	CACGACGTTGTAAAACGACGCCATCACATTCATGCACA	TTCAACTTCCCCAACACTCC
16	SSR 6425	CACGACGTTGTAAAACGACTGCTCAGTTCTGTGGTCCTG	TGGTTTATTCATCCAACATAGCA
17	SSR 6769	CACGACGTTGTAAAACGACGAACACGTGCCAACATAAAAGAAC	CTAAGATGTCGGCAGTTCTGTAAC
18	SSR 6671	CACGACGTTGTAAAACGACCAAACTTTGATATCGATCCTTG	GTTCTCTCATGCCATGATG
19	SSR 6774	CACGACGTTGTAAAACGACGAATCCACTCGTTTTAGAATCTC	GAGAGTGTTTTCAAGTGTGAACC
20	SSR 6777	CACGACGTTGTAAAACGACCGAAGCATGTGGACACGTAC	CATTGAACAAACATCGCTGAAGC
21	SSR 6800	CACGACGTTGTAAAACGACTGACTCTTTCTCTCAAGTTA	GATGGGTTGTGGAAAATAAA
22	SSR 6807	CACGACGTTGTAAAACGACGAACTATTATACAATCATGCACGA	GTAGCTTACTTCAATGATTAG
23	SSR 6819	CACGACGTTGTAAAACGACGCAACATCGAGGAAGATGCAAAG	CAAAAGAAATCATGATCTAACTTC
24	SSR 6844	CACGACGTTGTAAAACGACAGTTCTATCAGTATATTTTCATTT	ATTGTTAATTAGAAACCTAGCTGGG
25	SSR 6862	CACGACGTTGTAAAACGACGTTAGAGGTATGTGTAAGATG	GGCATTTCCATCCTCATCTC
26	SSR 6866	CACGACGTTGTAAAACGACTGGTGGGTTGGTATCGAAAG	GCAACCTTACGAAATCTCAAA
27	SSR 6924	CACGACGTTGTAAAACGACGATCACCTCCCACACCTCAG	TAGCAGTTTCCCACCAGCTT
28	SSR 6827	CACGACGTTGTAAAACGACTGACGGGATCTCTCAAGTTA	GATGGGTTGCCCAAAATAAA

### Gel electrophoresis

The amplification PCR products were analysed by electrophoresis on a 6.5% polyacrylamide denaturing gel on Licor 4300 sequencer (LICOR Inc., NE, USA). Before loading the gel, the multiplexed PCR products were denatured at 94° C for 3 min, and then the plate was placed on ice. The amount of denatured DNA loaded in the wells of the deposition rack was 2.5 μL. An infrared camera detected the fluorescence signals emitted by the marked fragments when excited with laser diodes at two different wavelengths (682 and 782 nm). The images were automatically recorded and downloaded for analysis. Allele sizes were estimated by comparing them with different bands of the size marker (ladder produced by CIRAD) [29].

### Scoring of bands and data analysis

All images of the gel profiles were printed for reading. A binary matrix was generated for all accessions based on the patterns of the bands observed at a particular locus. The GenAlex 6.4 software [30] was used to assess the genetic diversity and to assess the genetic differentiation (F_ST_) among populations. The statistical parameters such as total number of alleles per locus (Na), number of effective alleles per locus (Ne = Ne = 1/ ($\sum p_{i}^{2}$)), Shannon’s information index (I = -1* ∑(Pi*Ln (pi))), observed heterozygosity (Ho = number of heterozygotes/number of genotypes (N)), gene diversity (He = $1‐\sum p_{i}^{2}$), unbiased expected heterozygosity (uHe = (2N/(2N-1)) * He)) and fixation index (F = 1- (Ho/He)) were determined as described by Pagnotta [31] for each SSR locus and populations (regions). The polymorphic information content (PIC) values were calculated for each SSR locus as PIC = 1 − Σ (pi2), where pi is the frequency of the ith allele. The genetic distance (D) between populations were also computed using the Pairwise Population Matrix of Nei’s Unbiased Genetic Distance [32]. Further an Analysis of molecular variance (AMOVA) to test the degree of differentiation among and within the sources of collection of the cowpea accessions and a principal coordinate analysis was performed using GenAlex software and plotted using R [33]. The relatedness between accessions was estimated by ward’s minimum variance method and a dendrogram was built on the 70 accessions using the Analyses of Phylogenetics and Evolution (ape) [34] package implemented in R [33].

The population structure of the 70 cowpea accessions was established using the Bayesian clustering method in STRUCTURE version 2.3.2 [35]. The length of the burn-in period and Markov Chain Monte Carlo (MCMC) were set at 10,000 iterations. To obtain an accurate estimation of the number of populations, ten runs for each K-value were performed with K ranging from 1 to 10 [36]. Further, Delta K values were calculated, and the appropriate K value was estimated by implementing the method by Evanno et al. [36] using the STRUCTURE Harvester program [37]. We used the simulation with the highest log probability for ancestry analysis and then classified individuals in groups based on an ancestry coefficient of 0.55 or higher [29, 38].

Principal coordinate analysis was performed to represent the spatial distribution of individuals from different populations using the using GenAlEx 6.5 [30] and plotted using R [33].

## Results

### Genetic polymorphism of SSR markers

A total of 164 alleles were generated by the 28 markers across the 70 accessions. The number of alleles detected per SSR primer pairs varied between two (2) to fourteen (14), with an average of 5.86 alleles per loci. The lowest number of alleles per locus was detected for the markers SSR6217, SSR6774, SSR6311, SSR6243, SSR6671, and SSR6288. The highest number of alleles was recorded for SSR6800. A total of 18 rare alleles were detected in this study. The number of effective alleles per marker ranged from 1.21 to 6.44, with an average of 3.05 with the markers SSR6571 and the marker SSR6807 having respectively the lowest and the highest number of effective alleles respectively. For the SSR loci, polymorphism information content (PIC) representing a measure of the allelic diversity for a specific locus varied from 0.20 to 0.89 with an average of 0.58. Ten SSR loci (SSR6243, SSR6215, SSR6819, SSR6800, SSR6239, SSR6807, SSR6844, MA120, SSR6866 and MA113) exhibited PIC values higher than 0.70, indicating their usefulness in discriminating genotypes. The observed heterozygosity values ranged from 0.00 to 0.38 with an average of 0.07, and the major allele frequency varied from 16.17% to 89.06% (Table 3). This study has also revealed a number of rare alleles and unique alleles. The rare alleles represented near 11% of the whole detected alleles, with a total of 14 rare alleles and a total of 3 unique alleles were also detected. SSR6800, SSR6245 and SS6215 have respectively produced a unique allele for the accession Amélassiwa 3, Kampirigbène and Agnokoko (Table 3).

**Table 3:** The number of alleles per locus, major allele frequency, expected heterozygosity, observed heterozygosity and polymorphism information content (PIC) of the 28 SSR markers across 70 cowpea accessions.

Markers	Alleles			Number of effective alleles^b^	Major allele frequency (%)^c^	Expected heterozygosity^d^	Observed heterozygosity^e^	PIC^f^
	Number^a^ per loci	Unique	Rare					
SSR6421	3	0	1	1.99	55.70	0.43	0.00	0.50
SSR6246	3	0	1	1.45	81.20	0.28	0.01	0.31
SSR6217	2	0	0	1.86	63.60	0.43	0.02	0.46
SSR6323	3	0	0	2.10	57.10	0.46	0.02	0.52
SSR6769	6	0	0	4.95	26.07	0.65	0.02	0.80
SSR6425	4	0	0	2.36	57.73	0.56	0.02	0.58
SSR6774	2	0	0	1.87	63.30	0.46	0.01	0.46
SSR6777	3	0	0	1.70	73.71	0.41	0.01	0.36
SSR6311	2	0	0	1.94	59.00	0.47	0.08	0.48
SSR6862	5	0	0	2.68	56.41	0.56	0.02	0.63
SSR6243	2	0	0	1.69	71.29	0.38	0.03	0.41
SSR6215	11	1	1	5.91	30.45	0.79	0.34	0.83
SSR6924	4	0	1	1.91	68.74	0.46	0.01	0.48
SSR6671	2	0	0	1.26	88.48	0.20	0.03	0.20
SSR6819	10	0	1	6.66	23.37	0.75	0.00	0.85
SSR6800	14	1	3	8.17	21.34	0.83	0.01	0.88
SSR6245	3	1	0	1.70	71.29	0.38	0.05	0.41
SSR6304	4	0	1	2.09	59.51	0.49	0.03	0.52
SSR6288	2	0	0	1.52	77.94	0.34	0.38	0.34
SSR6239	9	0	0	6.04	24.03	0.78	0.02	0.83
SSR6807	13	0	1	9.21	16.17	0.83	0.08	0.89
SSR6241	7	0	0	2.47	61.20	0.58	0.32	0.59
SSR6844	11	0	2	6.69	25.76	0.81	0.02	0.85
MA120	10	0	1	5.58	26.65	0.77	0.03	0.82
SSR6866	11	0	0	7.14	28.42	0.80	0.00	0.86
MA113	12	0	3	7.83	23.71	0.79	0.00	0.87
SSR6289	3	0	1	1.25	89.06	0.19	0.19	0.20
SSR6827	3	0	1	1.63	74.69	0.38	0.27	0.39

^a^ Total (164), Average (5.86), Minimum (2), Maximum (14)

^b^ Average (3.63), Minimum (1.25), Maximum (9.21)

^c^ Average (52.71), Minimum (16.17), Maximum (89.06)

^d^ Average (0.54), Minimum (0.19), Maximum (0.83)

^e^ Average (0.07), Minimum (0.00), Maximum (0.38)

^f^ Average (0.58), Minimum (0.20), Maximum (0.89).

### Genetic relationship of cowpea populations

SSR markers used in this study revealed high percentages of polymorphic loci (average of 99.28%). The lowest percentage of polymorphism was observed for cowpea population one, corresponding to the Centrale region, while the percentage of polymorphism observed for each of the other four regions was 100%. The number of alleles detected in each population is not uniform and varied from 102 alleles in the cowpea population from the Centrale region to 127 alleles in the population of the Plateaux region. Among the five population investigated, the mean values of observed alleles (Na) and effective alleles (Ne) were 3.96 and 2.92, respectively. Population 3 (from Centrale Region) recorded the lowest value of Na (3.64), while the highest value (4.54) was recorded from population 5 (from Savane Region). For the effective number of alleles, the lowest value (2.85) was recorded from population 3 (from Maritime Region), while the highest value (3.07) was displayed by population 5. Shannon’s information index ranged from 1 to 1.08 with a mean of 1.03. The observed heterozygosity (Ho) ranged from 0.07 (population 5, population 4 and population 3) to 0.08 (population 1 and population 2) with an average of 0.07. The expected heterozygosity (He) was moderately high and ranged from 0.53 (Population 2) to 0.55 (Population 1 and Population 3), with an average of 0.54. The unbiased expected heterozygosity ranged from 0.56 (Population 2, Population 4 and Population 5) to 0.58 (Population 1 and Population 3), with an average of 0.57. According to the results, the five regions displayed almost similar diversity of cowpea. The values for the inbreeding coefficient expressed by the fixation index F ranged from 0.79 (Population 5) to 0.85 (Population 3) with an average of 0.82 at the population level (Table 4). Genetic similarity among the five populations was high and ranged from 0.85 between Population 1 and Population 4 to 0.94 between Population 3 and Population 4 (Table 5).

**Table 4:** Summary of different cowpea population diversity statistics averaged over the 28 SSR loci.

Population	NA	Allele number	% P	Na	Ne	I	Ho	He	uHe	F
**Centrale**	10	102	96.43	3.64	2.86	1.01	0.08	0.55	0.58	0.85
**Kara**	11	105	100	3.75	2.86	1	0.08	0.53	0.56	0.82
**Maritime**	14	111	100	3.96	2.84	1.05	0.07	0.56	0.58	0.85
**Plateaux**	15	110	100	3.93	2.94	1.03	0.07	0.54	0.56	0.81
**Savannah**	20	127	100	4.54	3.07	1.08	0.07	0.54	0.56	0.79
**Mean**	14	111	99.28	3.96	2.92	1.03	0.07	0.54	0.57	0.82
**SE**	1.16	4.32		0.19	0.15	0.05	0.012	0.018	0.019	0.028

NA = number of accessions per population, % P = percentage of polymorphic Loci within each population, Population 1 = cowpea accessions from Centrale Region, Population 2 = cowpea accessions from Kara Region, Population 3 = cowpea accessions from Maritime Region, Population 4 = cowpea accessions from Plateaux Region, Population 5: cowpea accessions from Savannah Region Na = Number of different alleles; Ne = number of effective alleles, I = Shannon’s Information Index, Ho = observed heterozygosity, He = expected heterozygosity, uHe = Unbiased expected heterozygosity, F = Fixation index.

**Table 5:** Pairwise population matrix of Nei’s unbiased genetic distance.

Population 1	Population 2	Population 3	Population 4	Population 5	
1.000					**Population 1**
0.898	1				**Population 2**
0.857	0.900	1			**Population 3**
0.847	0.865	0.945	1		**Population 4**
0.849	0.874	0.901	0.922	1	**Population 5**

The genetic differentiation indices between populations (Fst) varied from 0.00 (between Centrale and Kara Regions, Kara and Savannah Regions, Maritime and Plateaux Regions) to 0.057 between Centrale and Maritime Regions. Differentiation appears to be null or low between accessions from different regions except for Centrale and Maritime Regions, which appears moderate (Table 6).

**Table 6:** Pairwise population Fst values.

Centrale	Kara	Maritime	Plateaux	Savannah	
0.000					**Centrale**
0.000	0.000				**Kara**
0.057	0.030	0.000			**Maritime**
0.024	0.010	0.000	0.000		**Plateaux**
0.012	0.000	0.039	0.028	0.000	**Savannah**

### AMOVA

AMOVA was performed using the matrix of distances for genetic differentiation. The results of AMOVA revealed that the majority of variance occurred within individuals and accounted for98% among individuals within regions of the total variation, whereas 2% and 13% of the variation was attributed to differences between populations. The results indicated that the diversity within regions (intra-regional diversity) was far greater than the diversity between regions (inter-regional diversity), and the low Fst value (0. 069) indicated a low genetic variation among regions. The haploid Nm was very high (8.970) indicating a high gene exchange among populations while the inbreeding coefficient and the overall fixation where respectively 0.830 and 0.835 across the SSR loci. These results demonstrated that genetic differentiation among subpopulations was low and within subpopulations was very high (Table 7).

**Table 7:** Analysis of molecular variance (AMOVA) based on 28 SSR markers.

Sources	df	SS	MS	Est. Var.	%
**Among Pops**	4	159.246	39.811	0.549	2%
**Within Pops**	65	2096.083	32.247	32.247	98%
**Total**	69	2255.329		32.796	100%
**Fixation indices**	Value				
**F**_**ST**_	0.069				
**F**_**IS**_	0.830				
**F**_**IT**_	0.835				
Nm	8.970				

df = degree of freedom; SS = Sum of squares; Est. Var = Estimated variance; % = percent variation; Fst = Fixation index; Fis = Inbreeding coefficient; Fit = Overall fixation index, Nm = gene flow

### Phylogenetic analysis and principal component analysis

**Fig 2:**
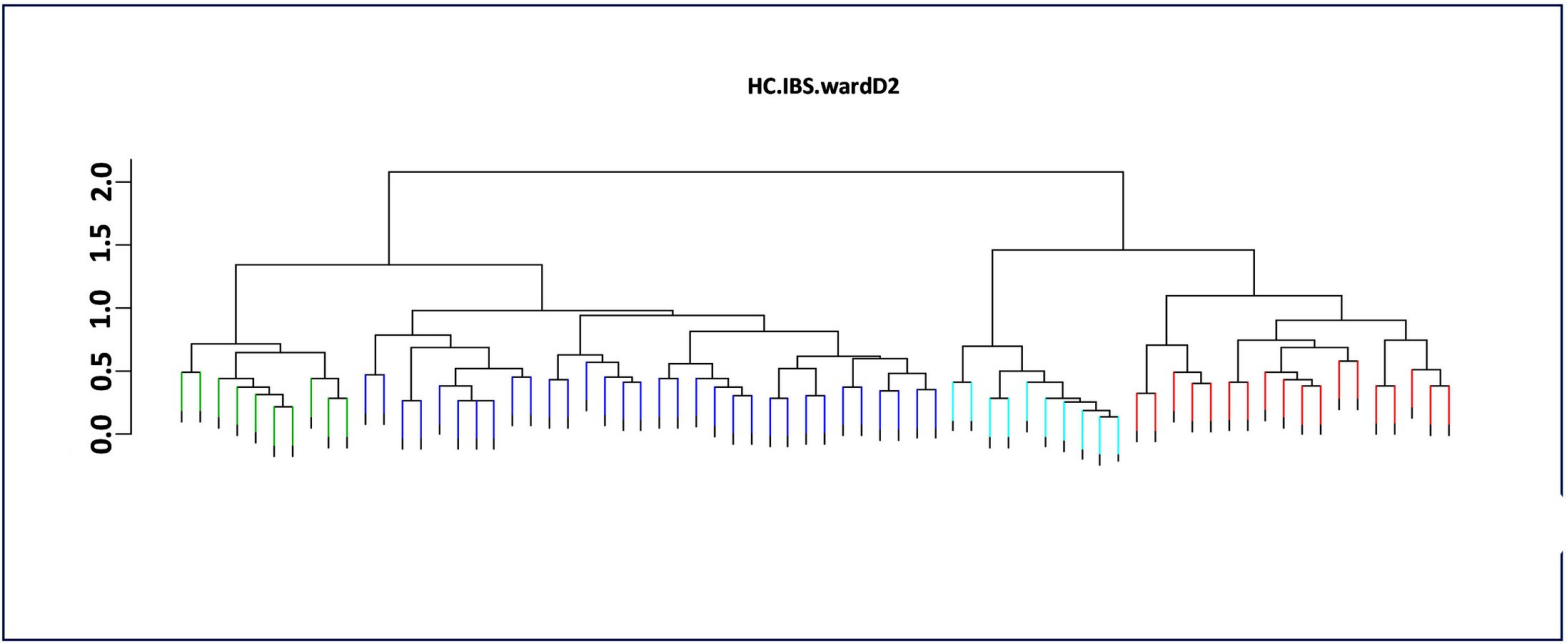
Phylogenetic tree among 70 cowpea accessions studied revealed by Ward’s minimum variance method.

The accessions studied were clustered into two main groups (I and II) based on Ward’s minimum variance method (Fig 2). There was no group made up exclusively of accessions from the same region and both groups can be further divided into two sub groups: sub groups A and B for group I and sub groups C and D for group II. Sub groups A and C were the smallest groups with 10 accessions in each of them Sub group A gathered a total of ten accessions collected from all the regions. All of the accessions included in that cluster except one are creeping-type accession. Sub group B was the largest group and contained 33 accessions with different growth habit (erected to creeping-type). It gathered 11 accessions from the Savannah Region (33.33%), eight accessions from the Plateaux Region, seven accessions from the Maritime Region, five accessions from the Kara Region and three accessions from the Centrale Region. Sub group C also gathered 10 belonging to the creeping-type with four accession from Kara region, 3 accessions from Savanes Region and 1 accession from each of the remaining regions while sub group D gathered 18 accessions from all the Regions except accessions from Kara Region.

The genetic distance among cowpea accessions in this study varied from 0.1432 between Kétséyi soukpélo an accession belonging to the erected-type with small purple grain collected from the Kara Region and Gouarga, an accession belonging to the creeping-type, with white small grain collected from the Savanes Region to 0.9317 between Hèkou hèkou a creeping-type accession with white medium size grain collected from Centrale Region and Kampirigbène a semi-erected type accession with white small grain collected in the Savanes Region (S1 Table).

**Fig 3:**
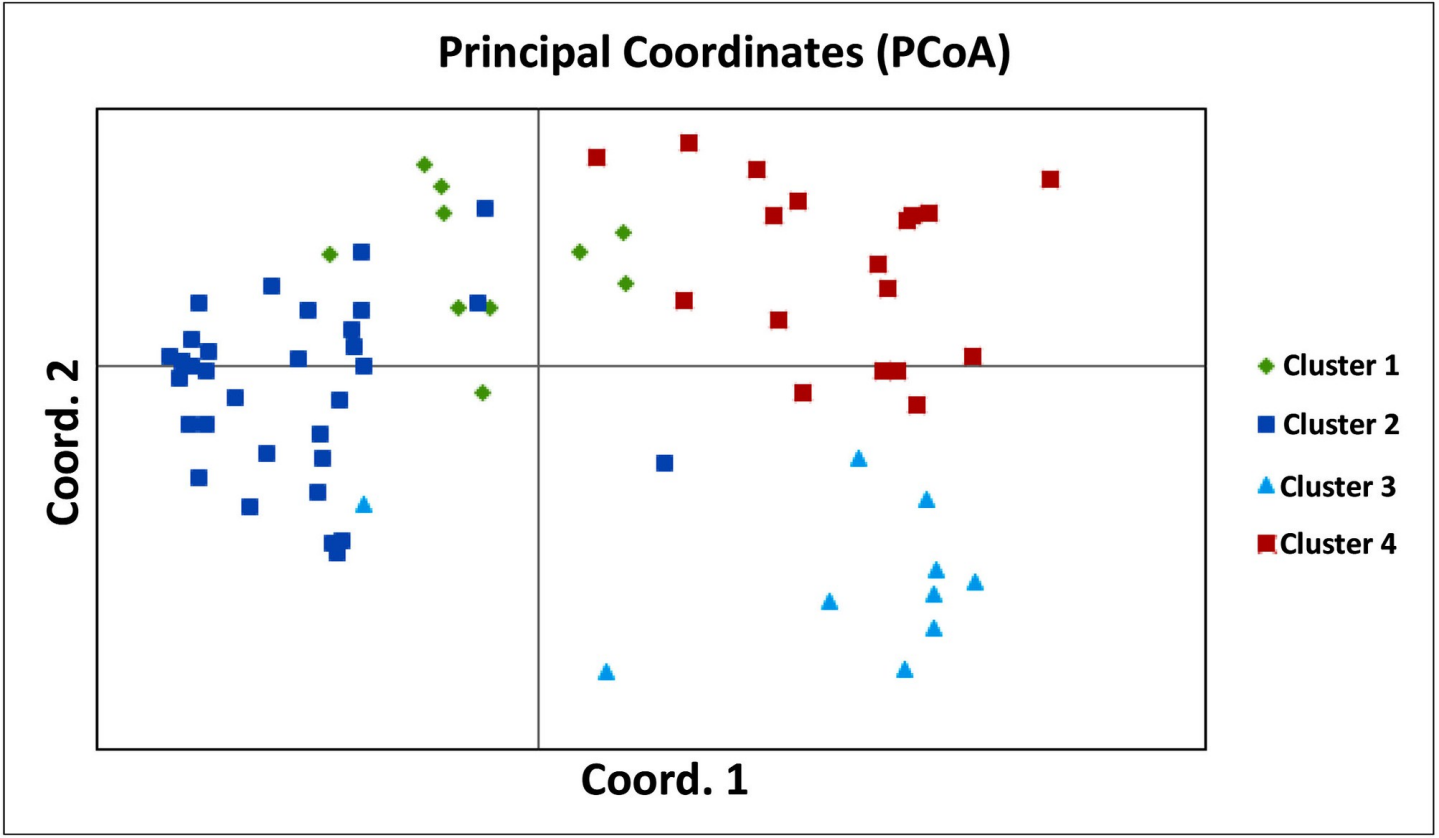
Principal coordinate analysis of four cluster of 70 cowpea accessions.

Genetic relationships among the selected accessions were further assessed using principal coordinate analysis (PCoA). The first three principal coordinates explained 10.62%, 17.73%, and 23.85% of the variance. The PCA further supported the grouping of the accessions in four groups (Fig 3).

### Population structure of the 70 cowpea accessions based on 28 SSR markers

**Fig 4:**
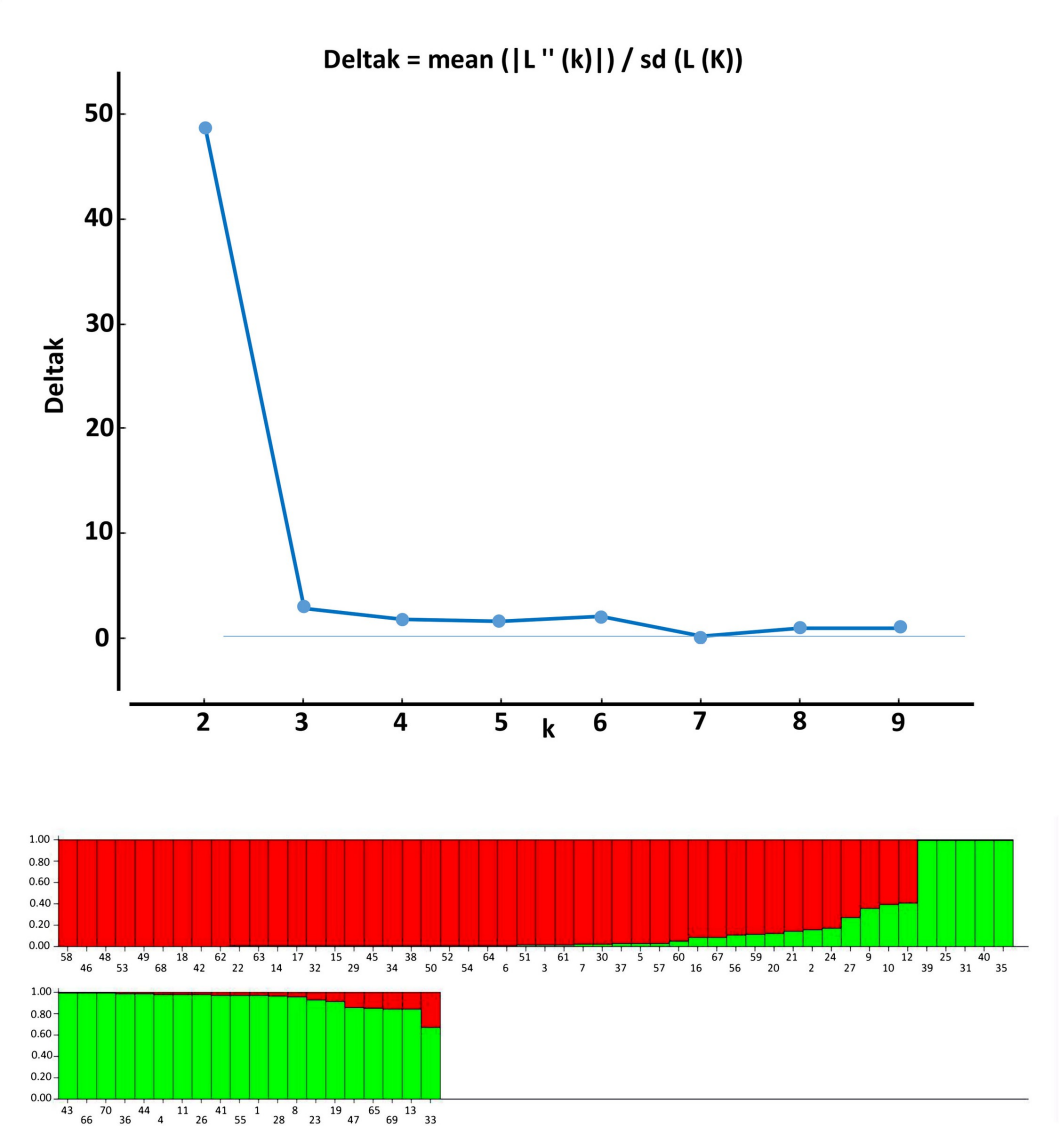
a. Graph of estimated membership fraction for K = 2, b. Population structure of 70 cowpea accessions (K = 2). The maximum of ad hoc measure ΔK determined by structure harvester was found to be K = 2, which indicated that the five populations could be grouped into two subgroups.

The structure analysis of the population structure based on the ΔK value grouped the seventy accessions into two subpopulations (clusters) (Fig 4A and 4B). Membership of all genotypes to a particular subpopulation was based on a likelihood threshold of 0.55. Subpopulation 1 had the largest membership with 64.28% of the accessions, while the smallest was Cluster 1 which only gathered 35.71% of the accessions (Table 8). Based on the threshold of 0.55, the study did not reveal any admixture among the accessions. Both subpopulations were composed of accessions from the five regions, and white-colored seeds dominated both. The first subpopulation comprised five accessions from the Centrale region, seven accessions from Kara region, nine accessions from Maritime region, 11 accessions from the Plateaux region and 13 accessions of the Savane region while subpopulation comprises five accessions from the Centrale region, fourth accessions from the Kara region, five accessions from the Maritime region, fourth accessions Plateaux region and 7 accessions of Savane region. Subpopulation 1 had more accessions from Savane and Plateaux regions, while population 2 had almost equal access to each region. Regarding the seeds coat color, subpopulation 1 was the most heterogeneous and included 66.67% of white-colored seeds, 11.11% of beige red-colored seeds, 8.89% of red wine-colored seeds and 4.44% of burgundy purple-colored seeds, while subpopulation 2 included 64% of white-colored seeds, 12% of beige red-colored seeds, 12% of purple -colored seeds and 8% of burgundy purple-colored of the reddish grey, golden yellow, blackish red and purple red colors were recorded respectively for one genotype in subpopulation one while the genotype presenting those seed color were absent in subpopulation 2.

**Table 8:** Genetic clusters and member of genotypes observed from population structure analysis of 70 cowpea genotypes.

Clusters	Genotypes	% Membership	He	Fst
1	Geno2, Geno3, Geno5, Geno6, Geno7, Geno9, Geno10, Geno12, Geno14, Geno15, Geno16, Geno17, Geno18, Geno20, Geno21, Geno22, Geno24, Geno27, Geno29, Geno30, Geno32, Geno34, Geno37, Geno38, Geno42, Geno45, Geno46, Geno48, Geno49, Geno50, Geno51, Geno52, Geno53, Geno54, Geno56, Geno57, Geno58, Geno59, Geno60, Geno61, Geno62, Geno63, Geno64, Geno67, Geno68	64.28	0.53	0.08
2	Geno1, Geno4, Geno8, Geno11, Geno13, Geno19, Geno23, Geno25, Geno26, Geno28, Geno31, Geno33, Geno35, Geno36, Geno39, Geno40, Geno41, Geno43, Geno44, Geno47, Geno55, Geno65, Geno66, Geno69, Geno70	35.71	0.54	0.15

% Membership = (number of accessions belonging to the cluster/Total number of accessions)*100, He = Average distances (expected heterozygosity) between individuals in same cluster, Fst = Fixation index inside each cluster

The expected heterozygosity (average distances between individuals in the same cluster) inside each cluster was average (respectively 0.53 and 0.54 respectively for cluster 1 and cluster 2) while the fixation index in both clusters was low (respectively 0.08 and 0.15 for cluster 1 and cluster 2). These results indicate a relatively low level of population structure (Table 8).

## Discussion

### Allelic pattern and gene diversity

An efficient evaluation of genetic resources can help reduce redundancies and build a core collection which can be screened to identify traits of interest. Further, a crop improvement and conservation program mainly depend on the presence of a genetic variability and on the characterization of the variability. Molecular markers are powerful tools for elucidating variations and relationships within and between crops germplasm populations. Among the genetic markers, SSRs are successfully applied in various breeding programs to study genetic diversity because of their multi-allelic nature, their level of polymorphism and the ease of their use [9, 20, 27, 29]. Previous studies have shown that SSRs are efficient markers for genetic diversity, population structure and QTL studies using cowpea germplasm [5, 39, 40]. Elucidating the genetic relationship in a crop germplasm depend on the relatedness of the germplasm being studied and on the informativeness of the markers used [41]. The 28 SSR markers used in this are considered as informative as they were chosen from the polymorphic microsatellite markers detected after screening of more than 300 cowpea markers available at Centre d’Etude Régional pour l’Amélioration de l’Adaptation à la Sécheresse (CERAAS) [29]. They are therefore suitable to assess the Togolese cowpea germplasm genetic diversity and to provide useful information for cowpea marker-assisted selection.

This study revealed a number of allele ranging from 2 to 14 allele per locus, which appeared to be relatively low compared to the value of 2 to 15 alleles per locus obtained by Sarr et al. [29] using 15 SSR markers to screen 671 cultivated cowpea from Senegal, the value of 1 to 16 obtained by Badiane et al. [27] by screening 22 local cowpea cultivars and inbred lines collected throughout Senegal using 44 SSR markers or the value of 2 to 17 obtained by Ali et al. [42] using 16 SSR to screen 252 cowpea accessions from Sudanese germplasm. However, the range of alleles detected by loci reported in this study is wider than those reported by other studies on cowpea germplasm diversity in Senegal (1–9), Ghana (1–6), Burkina Faso (5–12), and Nigeria (2–5) [2, 7, 9, 43]. Given the relatively lower number of alleles per loci reported by the latter cited studies, one might think that the cowpea germplasm they assessed was less diversified than the one used in this study. However, the observed difference might be explained by the difference in the number of accessions screened and the number of markers used. Lacape et al. [44] reported that the number of amplified alleles per locus depends on the selected markers and the type of germplasm. On these factors, the technique used for DNA separation during electrophoresis and the allele detection can be added.

The estimated average PIC value (0.67) recorded in the current study was similar to the value (0.68) reported by Ogunkanmi et al. [45], higher than the values reported by Asare et al. [9], Badiane et al. [27] and Ali et al. [42] who have respectively reported average PIC values of 0.38, 0.23, and 0.56. Therefore, SSR markers used in this study confirmed an interesting genetic diversity in the Togolese cowpea germplasm. Further, the study has detected ten SSR loci (SSR6243, SSR6215, SSR6819, SSR6800, SSR6239, SSR6807, SSR6844, MA120, SSR6866 and MA113) with PIC values higher than 0.70, indicating their usefulness in discriminating genotypes in future breeding programs.

The average gene diversity expressed by the expected heterozygosity (He), which is a measure of genetic diversity observed in the present study (0.54), was higher than the value (0.488) reported by Ali et al. [42] for a Sudanese cowpea germplasm and the value of 0.135 reported by Mafakheri et al. [46] in a study of 32 cowpea genotypes collected from different countries. It also appears to be higher than the value of 0.234 reported by Seo et al. [47] for 229 Korean accessions genotyped with the Cowpea iSelect Consortium Array containing 51,128 single-nucleotide polymorphisms (SNPs). The apparent moderate value of the expected heterozygosity can be explained by the diversity of cowpea farmer’s ethnics group in Togo. These ethnic groups have different criteria of selection when it comes to their preferred cowpea accessions. This difference in terms of criteria might have impacted the diversity of the crop in the country like concluded by Dagnon et al. [15] when studying the agromorphological variability of Togolese cowpea accessions. The observed heterozygosity (0.073) revealed by our study is low compared to He which means there is a deficit of heterozygotes in our population. Comparable results were obtained by Seo et al. [47] and by Fatakun et al. [48] when respectively studying a Korean cowpea germplasm and a set of 370 cowpea accession obtained from IITA’s Genetic Resources Center. Indeed, in their studies, the authors have respectively reported the values of 0.003 and 0.075 as an Ho overall mean and the values of 0.234 and 0.296 as He overall mean. This deficit of heterozygotes observed in our study is confirmed by the high value of the inbreeding coefficient (Fis = 0.830). These observations can be explained by the selection pressure exerted by farmers that might have reduced the polymorphism level in the cowpea population.

### Genetic relationships and population structure analysis

The dendrogram generated based on Ward’s minimum variance method has divided the 70 accessions in two main groups which is consistent with the results obtained from the population structure analysis. Each group can be further divided in two sub groups. This indicates the existence of a high degree of genetic diversity in the germplasm evaluated in this study. Therefore, these germplasms could serve as a valuable source for the selection of diverse parents for a breeding program aimed at creating new cultivars associating different traits of interest. However, in this study, the grouping of the accessions was not observed according to regional basis. Asare et al. [9] have also reported the same pattern when studying the genetic diversity and phylogenetic relationship among 141 cowpea accessions collected throughout the nine geographical regions of Ghana using SSR markers. Indeed, their accessions were clustered into five main branches, each of which was loosely associated with the geographical regions from which samples were obtained. The Principal Component Analysis (PCA) was performed to represent the spatial distribution of individuals from different populations and it further supported the grouping of the accessions based on Ward’s minimum variance method.

Like in many sub-Saharan African countries, cowpea accessions grown in Togo are identified by local names given by farmers based on their morphological characteristics such as seed characteristics, plant and pod shape [15]. This nomenclature can lead to a redundancy of some accessions in the germplasm collection. In this study, the highest genetic similarity (0.9317) was observed between two accessions having the same color but different growth habit and grain size. A great number of markers allowing a wider genome coverage will be more adequate to establish the exact relationship among the accessions.

The population structure analysis based on STRUCTURE software revealed the presence of two subpopulations among the 70 cowpea accessions collected from the five regions of Togo, while Sarr et al. [29] and Xiong at al. [38] reported three populations when they respectively studied the genetic structure of 671 cultivated cowpea accessions from Senegal and the population structure of 768 cultivated cowpea genotypes from the USDA GRIN cowpea collection, originally collected mainly from around the world. Two main populations were also reported for the Korean cowpea germplasm. However in the case of Korean germplasm, another peak was observed at K = 3, suggesting the accessions were further dissected into three populations [47]. The result of the population structure analysis tends to support the result of the phylogenetic analysis as it also gathered our accessions in two regardless their geographical zone of collection.

In the present study, the genetic variation components confirmed fair genetic diversity among individuals within regions (98%) than among regions (2%). The current study agrees with the findings of Sarr et al. (2020), who also reported a higher percentage variation among individuals within regions (75%). However, the percentage of variation attributed to differences between populations obtained in their study is higher than the 2% obtained in the current study. As already suggested by Sarr et al. [29], the high intra-regional diversity could be linked to the presence of many different accessions in each region. While the low genetic diversity between regions could be partly explained by the distribution of the same cowpea seed (same accessions are found everywhere) in all the regions through donations, seed companies, or agricultural extension services. This is confirmed by the high gene flow (Nm = 8.970) value which is a sign of high gene exchange among populations. Accessions from Kara seem to be close to the Savannah and Centrale region, given the zero value of the differentiation indices between the Kara and Centrale regions. Indeed, relatively low genetic distance were observed between some accessions of those regions. It is for example the case between the accession named Kadjarga form Kara Region and the two accessions Malgbong bomoine and Malgbong bopiel from the Savanes (Nei’s GD respectively equal to 0.20 and 0.29) (**S5 Table**). The observed similarity can be explained by the proximity of Kara Region to the two other regions. In fact, Kara Region is located in-between those two regions. This proximity might favor exchange of seeds between farmers of these regions.

The value of Fst was observed to be 0.069, indicating little differentiation among populations. The fixation index (Fst) obtained in the current study was much lower than the value of 0.114 obtained for the Senegalese germplasm and the value of 0.16 obtained by Fatokun et al. [48] when studying cowpea mini-core collections obtained from the IITA’s Genetic Resources Center. This lower fixation index supports the higher He value (He = 0.54) obtained in this study compared to the values obtained in the aforementioned studies (He between the range of 0.389 to 0.480 for the Senegal germplasm and mean He = 0.292 for the IITA mini-core collection). The inbreeding coefficient detected in our germplasm is high (Fis = 0.830) but is in the range of the value of 0.746 and the value of 0.988 obtained by Fatokun et al. [48] and Seo et al. [47] when respectively studying 370 cowpea accessions obtained from IITA’s Genetic Resources Center and 235 Korean cowpea accession.

## Conclusion

In Togo, cowpea is one of the main legume crops. However, the crop is poorly characterized. The current study provides useful information on the variability of SSR markers leading to a better understanding of the population structure and the genetic basis existing. It is the first study to address the genetic characterization of the Togolese germplasm, and it showed that the genetic structure does not depend on regions. The results obtained from this study will serve as basic information by providing options to breeders to develop, through selection and breeding, new and more productive cowpea cultivars that are adapted to changing environments. Furthermore, the collected germplasm could also be used for developing population for QTLs mapping studies in order to identify loci controlling traits with agronomic importance.

## Supporting information

S1 Table SSR markers position on cowpea chromosome. (XLSX)

S2 Table Binary matrix generated from the gel analysis. (XLSX)

S3 Table Input files for GenAlex analysis. (XLSX)

S4 Table Input file for PCA analysis. (XLSX)

S5 Table Nei’s genetic distance. (XLSX)
